# Short term global health experiences and local partnership models: a framework

**DOI:** 10.1186/s12992-015-0135-7

**Published:** 2015-12-18

**Authors:** Lawrence C. Loh, William Cherniak, Bradley A. Dreifuss, Matthew M. Dacso, Henry C. Lin, Jessica Evert

**Affiliations:** Dalla Lana School of Public Health, University of Toronto, 155 College Street, Sixth Floor, Toronto, Ontario M5T 3M7 Canada; Brooklyn, NY USA; Department of Family and Community Medicine, Markham-Stouffville Hospital, University of Toronto, 381 Church Street, PO Box 1800, Markham, ON L3P 7P3 Canada; Bridge to Health, 491 Lawrence Avenue West, Suite 301, Toronto, ON M5M 1C7 Canada; Department of Emergency Medicine, University of Arizona College of Medicine, 2800 E Ajo Way, Tucson, AZ 85714 USA; Global Emergency Care Collaborative, PO Box 4404, Shrewsbury, MA 01545 USA; University of Texas Medical Branch-Galveston, Center for Global Health Education, 301 University Blvd, Galveston, TX 77555 USA; Children’s Hospital of Philadelphia, 3401 Civic Center Blvd, Philadelphia, PA 19104 USA; Brooklyn, NY USA; Department of Family and Community Medicine, University of California San Francisco, 500 Parnassus Avenue MUE3, San Francisco, CA 94143 USA; Child Family Health International, 995 Market Street, Suite 1104, San Francisco, CA 94103 USA

## Abstract

Contemporary interest in in short-term experiences in global health (STEGH) has led to important questions of ethics, responsibility, and potential harms to receiving communities. In addressing these issues, the role of local engagement through partnerships between external STEGH facilitating organization(s) and internal community organization(s) has been identified as crucial to mitigating potential pitfalls. This perspective piece offers a framework to categorize different models of local engagement in STEGH based on professional experiences and a review of the existing literature. This framework will encourage STEGH stakeholders to consider partnership models in the development and evaluation of new or existing programs.

The proposed framework examines the community context in which STEGH may occur, and considers three broad categories: number of visiting external groups conducting STEGH (single/multiple), number of host entities that interact with the STEGH (none/single/multiple), and frequency of STEGH (continuous/intermittent). These factors culminate in a specific model that provides a description of opportunities and challenges presented by each model.

Considering different models, single visiting partners, working without a local partner on an intermittent (or even one-time) basis provided the greatest flexibility to the STEGH participants, but represented the least integration locally and subsequently the greatest potential harm for the receiving community. Other models, such as multiple visiting teams continuously working with a single local partner, provided an opportunity for centralization of efforts and local input, but required investment in consensus-building and streamlining of processes across different groups.

We conclude that involving host partners in the design, implementation, and evaluation of STEGH requires more effort on the part of visiting STEGH groups and facilitators, but has the greatest potential benefit for meaningful, locally-relevant improvements from STEGH for the receiving community. There are four key themes that underpin the application of the framework:

1. Meaningful impact to host communities requires some form of local engagement and measurement
2. Single STEGH without local partner engagement is rarely ethically justified
3. Models should be tailored to the health and resource context in which the STEGH occurs
4. Sending institutions should employ a model that ultimately benefits local receiving communities first and STEGH participants second.

Accounting for these themes in program planning for STEGH will lead to more equitable outcomes for both receiving communities and their sending partners.

## Background

Short-term experiences in global health (STEGH) abroad are becoming increasingly popular among healthcare trainees and practitioners [1, 2]. A ever-growing contemporary number of organizations based in high-income countries (HICs) offer various STEGHs to low and middle-income settings (LMICs) which vary in length, from weeks to months, as well as purpose, be it educational, research, or community service. Taken together, STEGH attract large amounts of funding and mobilize thousands of volunteers and trainees each year [3].

Over the past 60 years, the implementation of the international development agenda has become a shared responsibility between governments, communities, the private sector, and civil society. Worldwide, non-governmental (NGO) and faith-based organizations (FBOs) contribute to a hundred billion dollar industry that plays a crucial role in development programming [4, 5]. In recent decades, academic institutions (such as medical schools and postgraduate medical education) have become increasingly involved in global health and development projects [6]. A variety of STEGHs thus occur within the present-day context of an unregulated amalgam of NGOs, faith-based organizations, and academic institutions.

Many STEGH rely on local organizations as hosts. Local partnership allows visiting groups to seek context-relevant community guidance with respect to their involvement. The literature increasingly identifies local partnership as an ethical principle around the conduct of STEGH, and outlines key considerations in such partnerships. Broadly, these call on STEGH institutions to:

- Avoid imposing additional resource burden on local partners
- Provide to local partners, funding commensurate to resources consumed
- Prepare written memoranda that outline the roles and responsibilities of each partner
- Ensure participation standards and expectations are clearly outlined by the local partner and community and
- Agree that shared responsibility sustainability, and capacity building must be the foundational basis of any engagement [7, 8].

Applying these ethical guidelines becomes more challenging when considering the variable nature of local contexts and partnerships involved in many of today’s STEGH. Certain very remote LMIC communities, for example, may receive one STEGH a year, partnered with a single local organization. Other LMIC communities, perhaps more easily accessible to sending organizations in HIC, might welcome multiple STEGH sending organizations annually.

This review examines different models of local partnerships employed by STEGH, and proposes a framework for categorization, outlining pros and cons of each model. Employing this framework is meant to allow sending and hosting organizations to consider their community context in assessing their current and desired partnership to support the conduct of impactful, locally-driven STEGHs.

### Elements of a community-focused framework of local partner engagement models

The framework was developed by consensus among the authors and collaborators representing various organizations that conduct STEGH. This group consists of five men and one woman from the Global North encompassing a diverse background of experiences and training in public health and preventive medicine, academics, development studies, family medicine, internal medicine, and emergency medicine. All authors hold primary or adjunct academic appointments at institutions based in the United States or Canada. The primary rationale for inclusion of these panel members was related to their leadership roles in non-profit organizations based in the United States and Canada actively working on the issues surrounding STEGH. Of note, one panel member reported close collaborations with a faith-based organization (FBO), which added an additional lens. As an initial effort examining these issues, the panel did not include STEGH partners from host communities abroad, though the aim is to include representative members in ongoing discussions striving toward balanced and diverse perspectives.

A cursory literature search was conducted to identify sentinel articles that would stimulate initial conversations. This keyword search of PubMed, completed in January 2014, employed the terms “global health”, “short term” and “partnership”, with resulting articles reviewed by the group and initial agreement reached on what constituted a relevant publication. These articles, together with the experience of the authors, were subsequently used in an iterative discussion process. Nearly a dozen discussions occurred via teleconference for approximately 30–60 min in length, with a majority of authors present for all meetings and all authors attending a plurality of meetings. Following these discussions, consensus emerged on three key descriptive framework elements for categorizing local STEGH partnerships, which were:

1. **Visiting partners:** the number and nature of visiting organizations from abroad working in the host community;
2. **Host community partners:** the number and nature of local partners in the host community, and
3. **Frequency/continuity of short-term visits** by the visiting organizations to the host community.

Definitions for these themes follow below. Discussions following agreement on these definitions aimed to identify various models of partnership engagement based on these themes, as well as identifying broad principles for framework application.

#### Visiting partners

This framework category considers visiting partners as any STEGH sending organizations working outside their frame of reference; their participants broadly “visiting” the LMIC community who is receiving and hosting the STEGH. Primarily examining the relationship between visiting STEGH groups and the local host from the visitors’ perspectives, this category also considers the total number of groups visiting as well as the nature of their work. As an example, too few visiting partners working in conjunction with a local partner may be less intrusive, but might also limit the impacts and robustness of external resources available for health development. Conversely, receiving too many visiting partners may overwhelm a local institution that lacks adequate structure and compensation, thus creating the potential to impose unintended burdens on local resources [9].

#### Host community partners

This framework category considers the perspective of host community organizations that partner with STEGH sending organizations. Even before the widespread dissemination of ethical guidelines calling for local partner leadership, some STEGH groups would partner with host community organizations to achieve shared goals, such as development of local academic institutions, NGOs, and/or FBOs. Partnerships might occur with single or multiple host community-based institutions. Partnerships between visiting STEGH organizations and multiple host community partners may increase resources through pooling to support a variety of development and health activities, which in turn could generate more significant population health impacts. However, multiple partnerships also presents the challenge of maintaining collaboration across often diverse stakeholders, priorities, and motivations. In contrast, a bilateral STEGH – local partner partnership may seem more limiting, focusing on a sole local partner potentially permits STEGH groups to cultivate a deep relationship with narrowly-defined but mutually beneficial goals.

#### Frequency of visits

This framework category address the time commitment that a visiting partner makes to its host partner(s). Panel members differentiated between whether a visiting partner has “boots on the ground” throughout the entire year on an intermittent or continuous basis. For definitional purposes, local staff hired by a foreign organization are considered members of the host community. Thus, a visiting partner that might employ local staff but only makes short visits once a year would be considered to be conducting intermittent visits. Continuous visits would be categorized if outside individuals are on the ground in the local community for a majority of time annually. It is important to note that this category aims to address only the continuity of presence of visiting partners, and does not ascribe comparisons with respect to valuing the work of visiting partners or local providers.

International partnerships require commitments of time, money, and resources. Early in the STEGH planning process, visiting partners must work with host partners to determine the scope of work, the available resources, and the community need they are addressing, and the impact that they hope to achieve. This will enable partners to consider either intermittent or continuous programming commensurate with their organizational strengths and weaknesses. These considerations should be constantly revisited as the partnership progresses.

### Applying the framework

Table 1 outlines these primary elements and the resulting categorization that unfolds. Each category is described briefly below.

**Table 1:** Framework for categorization of STEGH, by local partner engagement

Nature of visits		Intermittent STEGH	Continuous STEGH
Visiting partner	Host partner		
Single	None	Parachute	Multiple parachutes
Single	Single	Single host partner receives intermittent visit from single visiting partner	Single host partner receives continuous visits from single visiting partner
Single	Multiple	Multiple host partners receive intermittent visits from single visiting partner	Multiple host partners receive continuous visits from single visiting partner
Multiple	Single	Multiple visiting partners work intermittently with single host partner	Multiple visiting partners work continuously with single host partner
Multiple	Multiple	Multiple visiting partners link with multiple host partners for a stand-alone purpose	Multiple visiting partners continuously link with multiple host partners

#### Single visiting partner, no local partner

STEGHs that are arranged by a single visiting organization without a local community partner are often colloquially termed “parachute” programs. Historically, many STEGH have occurred in this manner. Groups of providers from HICs would spontaneously head off on short-term relief missions, either via a personal contact in a host community abroad for whom they did not have a long-term relationship with, or at random. Following the 2010 earthquake in Haiti, for example, many groups of well-intentioned individuals travelled to the country of their own volition to volunteer and provide services to people displaced by the crisis. These undertakings often occurred parallel to one another and official efforts, and were largely panned as poorly prepared and contributing to the chaos in the acute aftermath of the natural disaster [10]. In less emergent situations, however, parachute STEGH continue to occur—with increasing attention being directed to their unintended effects and the need for greater local partnership [8, 9].

#### Single visiting partner, single local partner, and intermittent STEGH

Responding to concerns, many STEGH sending organizations are transitioning to a model by which their programs are supported by a host partner on the ground; the most common resulting partnership thus occurs between a single visiting STEGH partner and a single host partner, with intermittent visits by the visiting organization. Such efforts are particularly common in the initial stages of a visiting partner’s involvement in a community, and when the community in question is more remote or has only begun to recently receive STEGHs.

In planning STEGH, the visiting partner (often an academic institution or NGO) relies heavily on the host partner to provide logistic support as well as guidance specific to the community context, particularly in feedback around planned programs being brought forward by the visiting team. In between the intermittent STEGH visits, however, any work in sustaining initiatives until the next visit falls to the host partner, while the visiting partner may provide external resource support and remote technical assistance or knowledge.

This partnership model limits the scope of work that can be accomplished by the visiting partner on STEGH, with a typical focus on more service-focused care or narrowly-focused research/educational initiatives that can be accomplished while they are “on the ground.”

#### Single visiting partner, single host partner, and continuous STEGH

For certain communities, the single visiting partner has a continuous presence on the ground, with staff and teams present in the community and in contact with the host partner at all times. This often takes place in the form of multiple STEGH, sent by the visiting partner, arriving in the community on a fixed schedule. Oftentimes, this model is adopted by particularly large and well-supported visiting partners with perhaps a longer-term interest in supporting health and development efforts in the community in question.

In an ideal application of this model, STEGH are part of a longer-term program undertaken between the visiting partner and the host partner. Each visiting STEGH, together with the host partner, provide an update and hand-off to incoming STEGH groups immediately following them. The host partner continues to oversee logistics, but in ideal situations, standardization of team compositions and programming allows some mitigation of the resource burden to their organization. Conversely, other versions of this model may simply mirror the nature of intermittent STEGH by visiting groups; in this case, STEGH groups from the visiting partner come continuously one after another to provide longitudinal impacts. In this situation, the focus of the host partner remains to provide logistic support and essential insight into the community.

Implemented well, a continuous presence has the potential to multiple impacts by redirecting efforts towards a longer-term, sustainable model. Simple continuous STEGH mirroring an intermittent model, however, has the potential to greatly increase the burden of work for the host community institutions.

#### Multiple visiting partners, single host partner, intermittent STEGH

In more established STEGH receiving community settings this is an extremely common model. A typical example is a mission hospital in an LMIC community that receives a number of STEGH from multiple unique visiting partners on a sporadic and intermittent basis. Commonly, groups that might be received over a defined period of time could include students from an academic institution in a HIC; volunteer groups from an NGO on a service experience; and STEGH from visiting FBOs from HIC.

In the most basic variation of this model, each visiting partner effectively has a single partner – single host intermittent relationship with the host partner in question. For the most popular communities, this is a not uncommon situation, given that funding might come from multiple various partners to support a plethora of programs. Typically, as knowledge of a STEGH-hosting community increases, its ability to attract STEGH similarly increases, and many host partners may find themselves engaged with a number of visiting partnerships.

In practice, this leads to significant resource burden on the part of the host partner. STEGH may arrive at the same time and there are now potentially multiple different projects or competing demands for the host partner to navigate. The resulting context presents challenges for impactful outcomes, given the enormous potential for duplication of effort and redundancy. Adding to these concerns around this model of partnership is that the nature of work undertaken by each individual STEGH is still limited by their intermittent presence. Essentially, at worst, these are essentially multiple single-visiting partner STEGH that might have a narrow focus on downstream, episodic care, with similar intended impacts but a much more significant resource burden to the host partner and community.

#### Multiple visiting teams/organizations, single local partner, and continuous STEGH

As described in the previous section, the arrangement of STEGH by visiting partners independent of one another and on an intermittent basis with a single host partner results in limitations to STEGH outcomes, particularly around effectiveness and sustainability. With expanded, collaborative partnerships, however, disparate visiting partners could emulate a more continuous model, linking and pooling each of their intermittent STEGH into a continuous, coordinated presence. This continuous presence eases the burden of host partners, particularly around advising and logistics support, allowing them to take on a more strategic role in guiding STEGH and truly collaborative programs that could arise.

While these potential benefits are evident, bringing multiple visiting partners together in conducting continuous STEGH contains additional complexities from the corollary continuous single visiting partner – single host partner partnership. Obvious potential differences include ideology (e.g., between an academic institution and a faith-based organization), motivation (e.g., some visiting partners with a service focus versus others with an educational focus), and preparation (some partners may undergo extensive training while others might be poorly prepared.) Successful employment of this model relies heavily on extensive discussions towards consensus and privileging the leadership and direction of the host partner.

#### Single visiting partner, multiple host partners, and intermittent STEGH

This model involves a single foreign organization sending one team to a variety of local sites for STEGH or coordinating efforts with multiple local stakeholders within receiving communities. Most typically, the community settings where this might occur are with visiting partners with a very narrow or specialized programming focus, or large with a diverse mandate and significant resources. For specialized visiting groups, their narrow focus allows them to quickly replicate their programs with local partner support. One good example is the mobilization of relief teams in situations of great need, such as humanitarian interventions. The intermittent nature of STEGH usually involves a relief team working in coordination with multiple host partners to deliver emergency/disaster mitigation measures.

For larger partners with a diverse mandate and significant resources, one could consider a visiting partner such as an academic institution with multiple departments that might conduct complimentary efforts in LMIC community. One department may establish a partnership with one host partner relevant to their mandate; another department might then be interested in establishing a STEGH program in the same community, but may partner with another host organization that is more in line with their mandate. The result brings the visiting partner together with multiple host partners, which provides broad opportunity for community impact through diverse STEGH, but also poses challenges around coordination and visiting partner messaging/branding, particularly if visiting partner internal communication processes are limited.

#### Single visiting partner, multiple host partners, continuous STEGH

A partnership model in which a single organization works continuously alongside multiple local partners actually often exits the STEGH realm, given the long-term commitment and dedication required. Groups that successfully coordinate multiple local stakeholders on a continuous basis can create meaningful community planning dialogue that leverages expertise, provided they remain committed to accurately representing potentially competing local needs.

This partnership model has the most potential to impact lasting changes in community context; in turn, many of the visiting partners that undertake such efforts are well resourced, well-staffed and well financed. Many of these visiting partners may have a brand or reputation that enables them to easily interact with leading stakeholders (e.g., local ministries of health) in accessing existing health systems. They may provide funding (particularly from STEGH participant fees or grant funding) to provide economic support for host partners and community programs. In turn, host partners provides strategic direction for programs and collaboration with impact assessments.

The challenges with this approach usually concern the competition for resources among the multiple hosting partners, particularly if there is a paucity other visiting partners in the community. The unintended impact of the visiting partner might be to act as an external pressure on hosting partners to alter their mandate, operation, or scope to align more closely with the priorities of the visiting partner. This has implications in that the visiting partner’s STEGH may end up not addressing actual community needs, but rather the needs that they are perceived to be visiting the community for. The principle of sustainability is thus even more important in a setting like this; the visiting partner may be able to bring STEGH to address immediate needs, but the focus should be on medium and long-term capacity building such that the many host partners are eventually able to transition into roles as the primary program or care provider.

#### Multiple visiting teams/organizations, multiple local partners, and intermittent or continuous

These specific models lie outside the realm of STEGH, but are relevant to the wider field of discussion around global health and development. In general, programs that mobilize multiple visiting teams to work with multiple local partners on an intermittent basis are rare and would likely fall into one of the other categories already described.

#### Broad principles in application

From the consensus discussions around various models described, the panel members identified four key principles to consider in the application of this partnership framework:

1. Meaningful impact to host communities requires some form of local engagement and measurement
2. Single STEGH without local partner engagement is rarely ethically justified
3. Models should be tailored to the health and resource context in which the STEGH occurs
4. Partners should employ a model that ultimately benefits local receiving communities first and STEGH participants second.

The second principle bears further explanation, in that literature increasingly highlights the potentially negative aspects of STEGH on host communities. These include lack of cultural competence, culture shock and insensitivity, the opportunity costs for local communities, and issues with continuity, particularly around funding and resources. Engaging partnerships has been proposed by several authors as a means to mitigate potential power imbalances and cultural clashes, establish longer-term resource transfers, and ensure relevance of STEGH work to community priorities [1, 3, 6].

Finally, measuring the impact of STEGH (as described in the first principle) is crucial [10]. Moving beyond good intentions, the discipline of global health requires the use of evidence to quantify and qualify impacts [11]. While many impacts remain intangible, there is increasing inquiry into the impacts of trainees involved in STEGH revealing benefits that go beyond community health [12]. A variety of methologies and approaches are relevant for STEGH including community-based participatory research (CBPR), implementation science, health impact assessment, and collaborative partnership evaluation tools [13-17].

## Conclusions

This taxonomic framework examines the local perspective around visitor-host partner relationships and STEGH. Its applicability lies with many potential groups involved in the conduct of STEGH, including academics, potential volunteers, and organizations in LMICs partnered with STEGH visiting groups.

Beyond the simple descriptions provided by this categorization, it is recognized that STEGH work is multifaceted and that the efficacy of each model will decrease or increase based on the degree of locally relevant considerations. In addition, we recognize that many of these models may occur on a continuum; for example, an initial “parachute” STEGH may be a portal into the development of a meaningful local partnership that will ultimately have the same considerations as some of the other models described in the framework. It is also important to remember that any of these models can provide community benefit if the described challenges are carefully monitored and addressed. This could be resource support for host community organizations negotiating between intermittent STEGH, or careful consultation of host partners by visiting partners conducting multiple, continuous STEGH before implementing a common project addressing a locally-identified need. Regardless of the model adopted, however, an earlier identified key discussion theme reminds us that STEGH must aim to tailor interventions and programming to the needs of the local partner in the host community, and not the visitors’ perceptions. As a first step, this review framework aims to present different models of partnership around STEGH to add to discussions about the importance of using local partner engagement to minimize community harms and optimize potential outcomes of STEGH being conducting in LMICs. Contemporary thinking, in applying various lenses of social justice, equity, and ethics, has encouraged a paradigm shift away from the model of the single visiting organization without a host partner. By focusing on community engagement and local partnerships, visiting partners are not only multiplying their potential impact, but are also designing programs that are informed by principles of ethics and social justice. The underlying intention is for STEGH-sending organizations to recognize their roles as visiting partners in the communities they serve, and to use this framework to evaluate their work. Evaluating partnerships will also allow these groups to improve their STEGH in ensuring their responsible conduct and in achieving desired host community outcomes of improved health and wellbeing. There is great potential for STEGH to accomplish meaningful work, but this will almost certainly require successful partnerships with host organizations and communities.
